# Effective Medical Waste Management for Sustainable Green Healthcare

**DOI:** 10.3390/ijerph192214820

**Published:** 2022-11-10

**Authors:** Sang M. Lee, DonHee Lee

**Affiliations:** 1 College of Business, University of Nebraska-Lincoln, Lincoln, NE 68588, USA; 2 College of Business Administration, Inha University, Incheon 22212, Korea

**Keywords:** medical waste management, priorities of medical waste management activities, hospitals, sustainable healthcare service, analytic hierarchy process

## Abstract

This study examines the importance of medical waste management activities for developing a sustainable green healthcare environment. This study applied a multiple methodological approach as follows. A thorough review of the literature was performed to delineate the factors that have been explored for reducing medical waste; hospital staff who handle medical waste were surveyed to obtain their opinions on these factors; the analytic hierarchy process (AHP) was applied to determine the priorities among the identified key factors; and experts’ opinions were consulted to assess the actual applicability of the results derived by the AHP. The study identified the following factors as the most important: medical waste management (26.6%), operational management issues (21.7%), training for medical waste management procedures (17.8%), raising awareness (17.5%), and environmental assessment (16.4%). This study analyzed the contributing factors to the generation of medical waste based on the data collected from medical staff and the AHP for developing a sustainable green healthcare environment. The study results provide theoretical and practical implications for implementing effective medical waste management toward a sustainable green healthcare environment.

## 1. Introduction

The impacts of the global COVID-19 pandemic on people’s daily life, the society, economy, and the environment involve trade-offs in many aspects. Technological innovations (e.g., rapid testing, tracking infected persons, online-based remote work and education, etc.) have been effective in preventing the spread of the pandemic. On the other hand, they also have drawbacks, such as waste treatment issues with the increased use of disposable products and inequalities due to social and digital divides. In particular, the increased volume of plastic waste due to COVID-19-related practices has significant ramifications that pose challenges with respect to ensuring a sustainable environment [1,2].

Penga et al. [3] predicted that 193 countries worldwide would generate an additional 8.4 million tons of plastic waste due to COVID-19-related activities, a 10% increase from the baseline since the World Health Organization (WHO) declared the disease a global pandemic in March 2020. Of the additional plastic waste generated during the pandemic, approximately 87.4% was discharged from healthcare institutions, including personal protective equipment (such as masks, sanitary gloves, and face shields), online packaging materials (due to increased online shopping), and virus test kits, accounting for 7.6%, 4.7%, and 0.3%, respectively. Geographically, waste generation was the highest in Asia (46.3%), followed by Europe (23.8%), South America (16.4%), Africa (7.9%), and North America (5.6%) [3]. In a simulation study of the dynamics of COVID-19-related plastic waste, Peng et al. [3] predicted that 3800 to 25,900 tons of debris have been released into the sea. With approximately 280 million confirmed COVID-19 cases at the end of 2021, the volume of medical waste is likely to be approximately 11 million tons, with about 34,000 tons being released into the sea [4].

In South Korea, medical waste generated due to COVID-19 is classified as “quarantine medical waste” according to the “Wastes Control Act” of 1999, and includes most items used by healthcare workers in COVID-19 treatment institutions, such as screening clinics [5]. With the rapidly increasing volume of medical waste during the pandemic, waste treatment facilities in South Korea have struggled despite operating at full capacity [5]. Furthermore, because massive amounts of medical waste are routinely incinerated, its environmental impact is not tomorrow’s problem, but an urgent current issue. In addition, the consequences of delays in collecting and/or disposing of medical waste could threaten the health of patients, guardians, healthcare workers in hospitals, and community residents. Therefore, joint efforts of healthcare providers and local communities are necessary to develop an environmentally sustainable healthcare system. As climate change, air pollution, plastic waste, and medical waste threaten human health and environmental sustainability, establishing an eco-friendly medical system can provide a better ecosystem and potentially offer long-term benefits to human health [2,6].

Considering infectious diseases caused by environmental pollution, there is an urgent need to develop a healthier ecosystem. Healthcare institutions generally use disposable products to minimize infection while treating patients. This strategy seems logical to prevent the spread of COVID-19. However, only 15% of all medical waste is considered “hazardous waste” which may be infectious or toxic, whereas 85% of the hospital-generated waste is general and non-hazardous waste, comprising food containers, packaging, and medical supplies (i.e., gloves and masks, among others) used in the screening process for patients without contagious diseases [6,7]. Different and more cost-effective approaches can be used to reduce medical waste from healthcare institutions, such as appropriately sorting the discharged waste and promoting the use of systems that employ high-temperature/pressure and chemical processes to sterilize medical equipment and materials. Great Ormond Street Hospital in London saved approximately USD 120,000 in expenses by eliminating 21 tons of plastic waste through training employees on the use of disposable plastic gloves [6].

Several initiatives and studies have investigated various aspects of medical waste, including the Medical Wastes Act [8]; treatment methods and the current status of waste management [9,10,11,12,13,14]; knowledge, attitudes, and practices of medical staff with respect to medical waste e.g., [1,15,16]; and COVID-19-related medical waste e.g., [3,6,17]. However, limited research is available on the sources of medical waste (e.g., healthcare institutions). Environmental protection and cost reduction through medical waste reduction depend on the activities and actions of related organizations and medical staff on the front lines of medical waste discharge. In addition, developing plans to initiate a change through healthcare workers can help establish a foundation for creating an eco-friendly healthcare environment.

The purpose of this study is to propose an operational plan for the effective management and treatment of medical waste generated in hospitals. Irrespective of how optimal a system or policy may be, an effective medical waste management program should address the following: (1) identify activities that can be implemented by employees who are generating medical waste; (2) determine the priority among these various activities; and (3) define the support needed at the organizational level to implement these activities.

To accomplish the study objectives, a thorough review was undertaken on relevant previous studies on the approaches and factors that were explored for reducing and managing medical waste. Second, to apply the AHP to determine the importance of the identified key factors, a survey of 16 hospital staff with more than 3 years of experience in handling medical waste was conducted to obtain their opinions on these factors for a pairwise analysis. Third, the AHP was applied to determine the priorities among the identified factors. Finally, three experts in medical waste management were interviewed to gain additional insights about the results of AHP and their actual application feasibilities. The study results can be used as a framework for developing a sustainable green healthcare ecosystem.

This paper is organized as follows. Section 2 reviews the relevant literature on medical waste and sustainable medical waste management. In Section 3, research design is presented for identifying and assessing the importance of the key factors that contribute to the generation of medical waste. Section 4 provides the AHP results and the opinions of experts on application feasibility of the AHP results. Section 5 summarizes the results of the study, implications, limitations, and suggestions for future research.

## 2. Literature Review

### 2.1. Medical Waste

Healthcare services enrich and prolong people’s lives through health promotion and disease prevention and treatment. However, healthcare services generate a large amount of medical waste in the process; 20% of such waste poses health risks, such as infection and exposure to hazardous chemicals or radiation [18].

The World Health Organization [19] provided the guidelines for medical waste management in its report “Safe management of waste from healthcare activities”. In these guidelines, the WHO defined healthcare waste as “all the waste generated by healthcare facilities, medical laboratories, and biomedical research facilities, as well as waste from minor or scattered sources”. ICRC [18] added that “medical waste covers all wastes produced in healthcare or diagnostic activities”. The United States Environmental Protection Agency (US EPA) [20] defined medical waste as “a subset of wastes generated at healthcare facilities, such as hospitals, physicians’ offices, dental practices, blood banks, and veterinary hospitals/clinics, as well as medical research facilities and laboratories”. In Article 2, No. 5, of the “Wastes Control Act” of South Korea, medical wastes are defined as “wastes discharged from public health and medical institutions, veterinary clinics, testing and inspection institutions, and other similar institutions, such as parts and extracts of human bodies and carcasses of laboratory animals, which may cause harm to human bodies by infection or otherwise and need to be specially controlled for public health and environmental conservation”. Although international agencies present diverse definitions of medical waste, their guidelines commonly include “waste generated from healthcare facilities” [18,19,20]. Hossain et al. [11] defined health care waste as “all types of waste produced in health facilities such as hospitals, health centers, and pharmaceutical shops”. In this study, medical waste refers to the waste generated during patient treatment processes (see Table 1).

Medical waste can be classified as hazardous or non-hazardous (general) waste. While non-hazardous medical waste does not pose a specific hazard, hazardous medical waste can cause diseases and environmental hazards [19,21]. The WHO [7] classifies medical waste into eight categories: ‘infectious waste, pathological waste, sharps waste, chemical waste, pharmaceutical waste, cytotoxic waste, radioactive waste, and non-hazardous or general waste’. As listed in Table 1, although the definition of medical waste differs slightly between institutions and countries, its classifications and contents are similar. Table 1 provides a detailed summary of the separation and treatment of infectious medical waste by organizations, countries, and date.

**Table 1:** Medical waste classifications and related details.

Entity/Year of Enactment	Classifications	Details of Infectious Waste
WHO: “Safe management of waste from healthcare activities” report in 1992 [19]. Terminology: healthcare waste	· Hazardous healthcare waste: sharp waste · Infectious waste · Pathological waste · Pharmaceutical waste · Cytotoxic waste · Chemical waste · Pressurized containers · Radioactive waste · Non-hazardous or general healthcare waste	· Cultures or strains of infectious pathogens · Waste from surgery or dissection of an infectious patient · Waste from an infectious patient in an isolation ward · Waste from contact with an infectious patient during surgery · Waste from contacts with animals that were inoculated with pathogens or infected with infectious diseases
US: Environmental Protection Act in 1990 [22]. Terminology: medical waste	· Isolation waste · Cultures, stocks · Human blood, blood products · Pathological waste · Contaminated sharp items · Contaminated animal carcasses, body parts, and bedding	· Quarantine waste determined by the Centers for Disease Control and Prevention (CDC) · Media and strains associated with infections · Blood · Pathological waste (human tissue, etc.) · Waste that can cause injury · Laboratory animal waste
UK: Environmental Protection Act of 1990 [23]. Terminology: healthcare waste	· Hazardous healthcare waste · Waste that can cause infection	· Microorganisms or toxins that can cause disease in humans or other living organisms · Items contaminated with pharmaceuticals containing biologically active pharmaceuticals · Sharp items, bodily fluids, and materials contaminated with hazardous substances as stipulated in Directive 67/548/EEC
EU: European Waste Catalogue of 1992 [24]. Terminology: healthcare waste	· Biological waste (separable anatomical waste) · Infectious waste · Disposal of chemicals, toxic waste, or pharmaceuticals, including cytotoxins · Waste that can cause injury (injection needles, scalpels, sharply broken materials, etc.) · Radioactive waste (as specified in the radioactive waste directive)	· Waste from childbirth, as well as the diagnosis, treatment, and prevention of human diseases · Waste from the diagnosis, treatment, and prevention of animal diseases
South Korea: Wastes Control Act of 1999 [25]. Terminology: medical waste	· Quarantine medical waste · Hazardous medical waste: tissue, logistics waste, pathological waste, perishable waste, biochemical waste, and blood-contaminated waste · General medical waste	· Any waste generated from the medical practice for a person quarantined due to an infectious disease · Waste that may cause harm to the human body, such as infection · Waste containing blood, body fluids, secretions, or excretions

### 2.2. Medical Waste Management for a Sustainable Healthcare Environment

According to the WHO [7], 15% of all medical waste generated is hazardous. In high-income countries, 0.5 kg of hazardous medical waste is generated per hospital bed every day, whereas it is 0.2 kg in low-income countries. During the COVID-19 pandemic, medical waste generation has accelerated. According to the United Nations Environment Program [26], the volume of medical waste generated from medical facilities related to COVID-19 is 3.4 kg per person and approximately 2.5 kg per hospital bed each day worldwide. During the pandemic, China generated approximately 469 tons of medical waste per day [3]. Japan, India, and Indonesia generated 876, 608, and 290 tons per day, respectively [26], while South Korea generated 476 tons per day [27].

Hassan et al. [10] argued that medical waste problems are caused because of the lack of awareness and willingness on the part of healthcare employees and ambiguous policies and laws about proper management of medical waste. Hossain et al. [11] emphasized that inappropriate behavior of employees and improper disposal methods of medical waste in hospitals can increase serious health risks and environmental pollution due to the contagious nature of the waste. Therefore, healthcare institutions require an operational strategy to train stakeholders involved in medical waste generation to manage this critical problem.

Although previous research on medical waste management focused primarily on the treatment of hazardous waste, the emphasis has recently shifted to operational strategies on managing the disposal of all types of medical waste. The reason for this trend is that the safe handling and disposal of all medical waste is a key step to preventing potential hazards (disease or injury) and pollution of the environment [9]. Although the transmission of blood-borne viruses and respiratory and other infections through inappropriate medical waste disposal has yet to be explored completely [19], the potential risks to human health and the environmental issue are obviously high [15]. Thus, medical waste management is now regarded as a critical component of high-quality medical services [28]. This change is a result of reports which have demonstrated how environmental pollutants generated during waste treatment are threatening the in which we live ecosystem and human health. Penga et al. [3] claimed that over eight million tons of COVID-19-pandemic-related plastic waste had been generated globally, with more than 25,000 tons discharged into the sea. This could cause adverse long-term effects on the marine environment.

Windfeld and Brooks [8] suggested the need for a standardized classification method to educate medical workers in the efficient management of medical waste. Thakur et al. ([29], p. 357) presented six dimensions of medical waste management practices as ‘experience, relationship, environmental factors, technology and qualification, economic factors, and firm’s capabilities.’ Healthcare institutions should develop medical waste management plans which include the daily collection, processing, separation, and packaging of medical waste, as well as the implementation of regular monitoring and training programs [11,15,30,31]. The effective operation and maintenance of medical equipment and facilities can help prevent the frequent generation of medical waste. For example, the life cycle of medical equipment can be extended through proper maintenance. Therefore, the appropriate operation and maintenance require continuous management activities, such as personnel training and supply of appropriate materials and spare parts.

To create a sustainable medical environment through the reduction in and management of medical waste, an appropriate organizational culture must be developed, encouraging the participation of all stakeholders who partake in medical waste generation [1]. This also requires the involvement and cooperation of all stakeholders, including the various occupations/departments within the healthcare institution, as well as the collaboration of patients, guardians, subcontractors, and communities [32]. Healthcare institutions should develop an integrated approach for medical waste management [29,30]. Therefore, one specific department should not bear the complete responsibility for medical waste reduction; instead, these activities should be practiced by all hospital members throughout the course of their work. For instance, the department in charge of medical waste disposal should practice proper separation to prevent general waste from being included in medical waste. Healthcare departments should attempt to reduce emissions from infectious waste and single-use products. Through these general activities, healthcare institutions can reduce medical waste generation and related operating costs, thus developing a sustainable healthcare service environment.

### 2.3. Operational Strategies for Effective Medical Waste Management

A well-prepared action plan can reduce the amount of medical waste without decreasing the quality of medical services provided by healthcare workers. Kwikiriza et al. [16] emphasized that clinical staff need to be fully aware of their critical role in effective medical waste management, because they are the ones who sort the waste at the point of generation. They also suggested that non-clinical staff tend to have limited awareness and experience about the treatment, segregation, and/or knowledge of medical waste management. To implement appropriate measures or activities to reduce the generation of medical waste in their daily operations, healthcare providers should have accurate information about the volume of medical waste being generated by them. Reducing the volume of waste that requires treatment is an obvious approach to lower the cost of waste management and improve the operational efficiency of the organization. Efforts to identify and eliminate unnecessary waste generation sources can positively impact the efficacy of developing a sustainable healthcare ecosystem. Therefore, the efficiency of medical waste management can be improved through correct waste classification and sorting at the point of material use.

The Organization for Economic Co-operation and Development (OECD) introduced the sustainable materials management system, which promotes efficient resource management throughout the entire lifecycle of a resource based on existing waste-management-oriented policies [33]. The G7 Toyama Environment Ministers’ Meeting in 2016 introduced a resource efficiency policy for promoting the efficient use of resources for sustainable development [33]. To implement a resource recycling economy, Kim et al. [34] suggested the following approaches: (1) suppression of waste generation; (2) waste reuse; (3) promotion of waste recycling; (4) energy recovery; and (5) appropriate disposal. As these approaches imply, implementing the activities that can reduce medical waste should be focused on frontline healthcare workers. To identify in-hospital activities that can reduce medical waste generation, the flow of waste processing phases must first be examined. Table 2 shows the general flow of medical waste management implemented in healthcare institutions in South Korea, from the generation to the treatment process of medical waste.

As shown in Table 2, after medicine and medical supplies are stocked in the purchasing department, goods are distributed at the request of each healthcare department. Medical waste is generated from this point onwards. For instance, medicine and medical supplies are purchased based on care departments’ needs for operations and patient treatment. These supplies become medical waste when they are used, disposed of, or their expiration dates are passed. Although expired medicine (i.e., drug ingredients) may be hazardous, medical supplies, such as syringes, surgical gloves, and gauze, are classified as general medical waste. However, even though such expired medical supplies, not in contact with patients, are considered general medical waste, they are often discharged as infectious medical waste or mixed with infectious medical waste for convenience, increasing the volume of generated infectious medical waste. Therefore, reducing unnecessary infectious medical waste is possible if healthcare workers, such as doctors and nurses, are aware of the value of proper waste classifications, separation processes, and emission reduction benefits for medical waste.

Johannessen et al. [30] suggested guidelines for evaluating and improving medical waste management based on the standard for >50-bed facilities and those with fewer than 50 beds with respect to the current medical service situation. The WHO [35], through its National Healthcare Waste Management Plan Guidance Manual, suggested a set of factors that should be considered prior to developing a medical waste management plan. The detailed contents of these factors can be summarized as follows. The medical industry and environmental protection are closely related [1]. For example, healthcare institutions that operate emergency and in-patient rooms emit greenhouse gases throughout the day. Medical waste is landfilled or incinerated, resulting in air pollutant emissions and water pollution due to landfill leaching, constantly raising concerns over environmental protection issues. Although hospitals are fully aware of the importance of medical waste management, they tend to assign the responsibility to a designated department. However, medical waste management cannot be achieved based solely on the role and efforts of the department in charge. Thus, medical waste management strategies should include operational standards and classification, as well as plans for potential waste disposal issues and operational implementation plans. Furthermore, relevant information about the effect of medical waste management on hospital operating costs should be disseminated to all organization members. In this perspective, medical waste treatment requires operational and management strategies.

Kwikiriza et al. [16] suggested that the incorrect use of personal protective equipment during the treatment/transport process of medical waste may cause infection risks as well as occupational hazard problems. Medical waste is often infectious; therefore, it must be stored safely for a certain period. Hossain et al. [11] indicated that although the safe handling and disposal of medical waste require a seamless process from the initial collection step to the final disposal stage, improper management practices are often prevalent. These problematic practices are caused by a lack of awareness, effective control, appropriate legislation, and specialized staff [11,16]. Thus, safety protocols should be established to continuously monitor the process to prevent leaks or other hazardous consequences.

The majority of medical waste can be classified as general waste; therefore, a classification policy or manual should be developed for implementation. Previous studies have provided convincing evidence that medical waste has a direct negative impact on the environment [9,10,16]. As such, every healthcare institution should endeavor to minimize environmental pollution by complying with the relevant policies and laws while providing a safe medical environment. In addition, because medical waste management involves social, legal, and financial issues, relevant authorities and associations should provide regular education to healthcare workers on new regulations, research findings, or new technologies [11,12,15,16]. Hospitals should provide education and training programs on the importance and impact of environmental management on organizational efficiency and community safety [31]. The prevention of possible problems that may arise in medical waste management is possible through effective training on the risks of erroneous waste classification and disposal, operational procedures, and responsibilities involved in medical waste management.

## 3. Methodology

### 3.1. Analytic Hierarchy Process

The analytic hierarchy process (AHP), a method developed by Saaty [36], is an effective decision-making tool for problems with multiple and conflicting evaluation factors and multiple alternatives solutions. In the AHP, after stratifying the evaluation factors for decision-making and reconstructing the primary factors into sub-items (secondary factors), the importance of each factor is determined through a pairwise comparison between factors prior to obtaining the final solution. The AHP approach is widely used because it allows flexible decision-making based on an intuitive perspective, including objective and subjective factors [37].

In this study, the AHP was applied because it is well suited to decision-making for medical waste management issues that involve complex and sometimes conflicting operational activities. The AHP is a subjective approach that focuses on a specific issue; therefore, the judgment of experts with practical experience is more appropriate than that of a large sample size [38,39]. Several previous studies used sample sizes between four and nine e.g., [40,41]. On the other hand, other researchers employed sample sizes greater than 30 [42,43]. In applying the AHP, the general suggested number of respondents ranges from 4 to 30. Medical waste occurs at the various medical service encounter points. Thus, in this study, we tried to involve personnel at many service encounter points, resulting in 30 participants.

### 3.2. Identification of Key Medical Waste Management Factors

To identify important factors in medical waste management and treatment processes in hospitals, this study analyzed the measures that can effectively reduce medical waste and develop a practical assessment method based on the input from managers of medical waste at tertiary healthcare institutions in South Korea.

A preliminary questionnaire was prepared to develop the measurement items that represent the operational and treatment activities of medical waste. As a pilot study, the questionnaire was distributed to staff who had sufficient experience in medical waste management activities in five Korean general hospitals. Based on the respondents’ suggestions, the measurement items were refined for clarity and accurate understanding. The identified measurement items of medical waste management for pairwise analysis are shown in Table 3.

### 3.3. Data Acquisition Process

To ensure effective decision-making with the verified importance of factors by AHP, we executed several steps. First, the final questionnaire developed for pairwise comparison evaluations of measurement items used nine-point Likert scales to determine the importance of items [36]. Second, the AHP was applied to determine important factors for medical waste management. Third, three experts who were in charge of medical waste management in their hospitals were interviewed to discuss the AHP results and their practicality. In this paper, AHP was applied to perform the following: (1) simplification of the evaluation item structure, (2) comparison of evaluation results, and (3) presentation of operational efficiency measures through decision-making based on the evaluation results.

**Figure 1:**
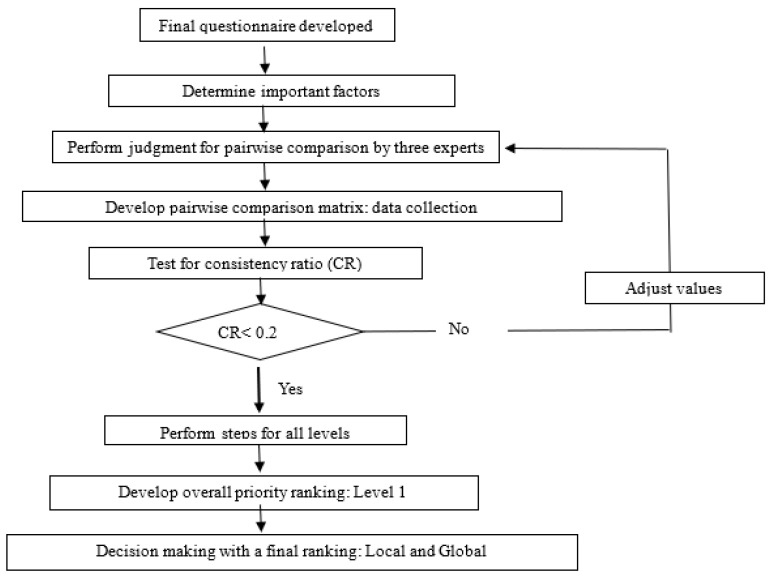
The analytic hierarchy process framework.

The ultimate goal of the application of AHP was to determine the priority of factors involved in medical waste management activities and treatment processes to secure a safe, waste-free environment. Figure 1 presents a schematic of the AHP framework employed in this study.

### 3.4. Data Collection

In this study, our survey respondents were restricted to healthcare workers with more than 3 years of experience in medical waste management activities (e.g., separating and disposing of wastes such as syringes, alcohol swabs, gloves, and general medical waste). Waste disposal workers at the hospital moved waste containers to a storage area first; then, they are transferred to an external treatment contractor.

For the AHP application, the survey was conducted during 10–25 January 2022, targeting 30 healthcare workers in hospitals with more than 500 beds. We received 23 responses (76.7%), although 7 were discarded due to incomplete items. Thus, the sample included 16 responses (69.6%). Table 4 presents the sample profile. Approximately 25.0% of respondents were from general wards, and the remaining 75.0% were from isolation wards, emergency rooms, intensive care units, and operating rooms in participating hospitals. The participants had knowledge related to medical waste at the following levels: high (50.0%), medium (37.5%), and low (12.5%). These results imply that the participants had a great deal of knowledge about medical waste. The proportion of respondents who participated in waste management training was high: 87.5%. The participants responded to the importance of medical waste management with the following activities (multiple responses): practice (100.0%), attitude (75.0%), and education training (25.0%).

## 4. Results

### 4.1. Consistency Test

To apply the AHP, a validity verification was first performed on survey items based on the consistency ratio (CR). Saaty [36] reported that a CR value of 0.1 or less is desirable, indicating that the probability of obtaining a logically impaired decision is less than 10%. When the CR value is ≤0.2, it indicates an acceptable range. In this study, the CR value was set to ≤0.2 based on the requirement of a pairwise comparison for each item [36]. The CR values for the five key items proposed in this study were all < 0.2; therefore, the criteria for decision-making in this study were satisfied. For the substitutability index, the opinions of respondents were not within the range of CR values due to the small sample size. A pairwise comparison matrix was analyzed using the geometric mean for the five factors that were considered most important in the management and treatment activities for reducing medical waste in healthcare institutions.

### 4.2. AHP Results

Table 5 shows the weights of five items and twenty detailed items used to prioritize important factors in managing medical waste based on the Expert Choice 2000 program. The results indicate that medical waste management (26.6%) is the most important factor for reducing medical waste generation, followed by operational management issues (21.7%), training for medical waste management procedures (17.8%), raising awareness (17.5%), and environmental assessment (16.4%). The interpretation of these analysis results is as follows.

First, medical waste management must be implemented safely with prescribed pro-cedures that should be executed by medical staff at contact points with medical waste to reduce its generation. The second priority factor to be considered is the operational issue of medical waste management (21.7%) such as standards and procedures. The third im-portant factor is training for medical waste management procedures (17.8%), indicating the need to provide a basic method easily accessible through education on medical waste management for healthcare workers or other organization members. Fourth is raising awareness (18.1%) about the impact of effective medical waste management. Reducing the volume of medical waste is only possible when the activities of the responsible depart-ments that generate waste are integrated into daily work activities, along with employee awareness of medical waste management. Finally, environmental assessments are neces-sary to understand the broad impact of medical waste on the medical environment.

First, medical waste management must be implemented safely with prescribed procedures that should be executed by medical staff at contact points with medical waste to reduce its generation. The second priority factor to be considered is the operational issue of medical waste management (21.7%) such as standards and procedures. The third important factor is training for medical waste management procedures (17.8%), indicating the need to provide a basic method easily accessible through education on medical waste management for healthcare workers or other organization members. Fourth is raising awareness (18.1%) about the impact of effective medical waste management. Reducing the volume of medical waste is only possible when the activities of the responsible departments that generate waste are integrated into daily work activities, along with employee awareness of medical waste management. Finally, environmental assessments are necessary to understand the broad impact of medical waste on the medical environment.

Table 5 also shows the results of the analysis on the local weights for each of the five evaluation items. Based on the analysis, for recognizing the importance of good healthcare waste management, raising awareness was the highest (31.8%), followed by setting up a waste management team with responsibility (25.7%), integration into daily operations (21.9%), and establishing a committee to develop a waste management plan (20.6%). These results indicate the importance of recognizing the significance of proper management and treatment activities for reducing medical waste generation.

For operational management issues, the items deemed important were in the following order: operational standards for medical waste items (35.2%), develop and implement a medical waste management plan (29.3%), medical waste management cost (23.1%), and plan for potential medical waste treatment problems (12.4%). The results show that the standards for medical waste management are most important among operational management issues. Thus, the establishment and execution of management plans are key factors.

For medical waste management, the following items were deemed most important: the safe storage of secure leak-proof and infectious medical waste (33.4%), policies or manuals on separation of medical waste by type (28.3%), simple-to-implement medical waste management for staff (including ancillary staff) (20.4%), and regular monitoring to ensure compliance with procedures (17.9%). Based on these analysis results, classification policies and manuals for each type of medical waste are imperative in medical waste management to reduce liability issues (criminal liability) after appropriate waste classification and disposal.

For environmental assessment, the important items were: a safe medical environment from medical waste (30.5%), environmental and health impact monitoring (29.3%), environmental management and training (22.7%), and policy, legal, and administrative frameworks (17.5%). Providing a safe medical environment is not only important for patients, but also for the members of the organization and local communities. From this perspective, a safe healthcare environment from medical waste was rated most important among the detailed items in the environmental evaluation. Infectious medical waste can cause secondary infections in hospitals, which might have also been reflected in the results. Regarding training for medical waste management procedures, the items deemed most important were: training on staff responsibilities and roles in managing medical waste (29.8%), training on waste separation operations (27.8%), education on the risks of incorrect medical waste management (23.5%), and technical training on the application of waste management practices (18.9%).

Organization members often do not have opportunities to interact with those in other departments. However, medical waste management is a special task which offers a shared goal for the benefit of all members of the organization. Thus, general education and training of all employees, in addition to those who are directly involved with the task, would be imperative to engage everyone in this effort.

Based on the analysis results for the 20 global evaluation items, there was no significant difference among the items. Safe storage of secure leak-proof and infectious medical waste (9.1%) was the highest, followed by simple-to-implement medical waste management for staff, including ancillary staff (8.7%), and operational standards for medical waste (8.4%).

### 4.3. Experts’ Opinions on the AHP Results

After the AHP results were obtained based on the responses of 16 medical workers in tertiary hospitals, we conducted interviews with experts in the related fields to derive additional insights from the study results. These interviews provided insiders’ perspectives on developing an effective implementation plan for medical waste management activities at the operational level. The different activity plans can also be delineated between the department in charge of waste management and supporting departments based on the experts’ ideas.

The three experts invited for the interview were selected among team leaders with more than 5 years of relevant work experience at tertiary hospitals in South Korea. Although each hospital has its own unique characteristics (e.g., operational structure, number of beds and employees, care units, etc.), there was no significant difference in their medical waste management programs among the hospitals of the 23 survey respondents. Some hospitals had their own dedicated medical waste management programs, whereas others had outsourcing arrangements with the municipal sanitation department. The hospitals that relied on the municipal sanitation program for waste management moved medical waste bins/boxes from each treatment room to medical waste storage areas. The collected medical waste was then transported and disposed of by contracted external firms. The departments in charge of medical waste management at these hospitals (e.g., general affairs or facilities departments) perform all necessary administrative procedures.

Table 6 summarizes the common problems, causes, and solutions suggested by the three experts. Based on both the AHP results and the experts’ opinions, medical waste management stood out as the first priority item. However, there was a difference in the second priority item. In the AHP results, the operational management issues item was rated as the second priority item. However, the experts rated training for medical waste management procedures item as the second priority. This may be due to differences in perspectives among managers (“provide education and training to staff to ensure proper sorting”) and staff involved in waste generation, handling, and sorting (“developing a manual for proper sorting of waste”). There was no significant difference among the priorities for the remaining items.

## 5. Conclusions

With the increasing concerns regarding contagious and infectious diseases, due to climate change as well as resistance to medications and treatments, the effective management of medical waste has become a strategic priority for healthcare providers. Packaging materials for medical devices are a recyclable resource. Medical waste, mainly incinerated for disposal, requires an eco-friendly treatment method to conserve the environment. Furthermore, healthcare institutions should properly classify and sort general hospital and medical waste in practice. The use of eco-friendly and low-risk containers is a constructive step in the classification and collection processes for medical waste.

This study analyzed the contributing factors to medical waste generation based on the data collected from medical staff and AHP for developing a sustainable green healthcare environment. The analysis results indicated the following priorities for the five key factors: medical waste management was rated the highest (26.6%), followed by operational management issues (21.7%), training for medical waste management procedures (17.8%), raising awareness (17.5%), and environmental assessment (16.4%). The analysis of local weights of the five factors revealed the following items as the most important: raising awareness—recognizing the importance of good healthcare waste management (31.8%); operational management issues—operational standards for medical waste (35.2%); medical waste management—safe storage of secure leak-proof and infectious medical waste (33.4%); environmental assessment—a safe medical environmental from medical waste (30.5%); and training regarding medical waste management procedures—training on staff responsibilities and roles in managing medical waste (29.8%).

### 5.1. Theoretical and Practical Implications

The results of this study have several important implications. First, practical medical waste management is the most important step in management and treatment activities for reducing the generation of medical waste. Medical waste is typically generated in each treatment unit and staff can discard it in the containers provided [10,16]. However, general waste, which does not require the same treatment as medical waste, is often misplaced into medical waste containers. Approximately 85% of medical waste is from general operations; hence, some of this may be reused or recycled [44]. Therefore, hospitals should implement action campaigns based on evaluations of what items can be reused or recycled to reduce medical waste generation.

Second, healthcare organizations should pursue qualitative improvements in the treatment of diseases for patients. From this perspective, hospitals are generally known as institutions that consume a high volume of single-use plastic products to minimize infections [45,46]. Different medicines and medical supplies are used in each department; therefore, detailed instructions or manuals on the handling of waste should be provided to healthcare workers for proper sorting and disposal to reduce the volume of generated waste.

Third, because awareness and education on medical waste management are important factors [10,11,16], all members of the hospital should be encouraged to participate in education on the value of medical waste management, especially resource circulation through the proper collection and separation of waste they generate daily. In other words, the generation of medical waste must be reduced to the greatest possible extent, minimizing the impact on the environment by reusing/recovering waste and establishing an eco-friendly green environment. In addition, medicines and supplies are used or become medical waste when their expiration dates are passed. Thus, it is important to manage inventories to avoid valuable medical supplies to become waste after the expiration dates. One way to reduce medical waste would be to include an effective inventory management program in employee education and training courses.

Fourth, medical waste management is subject to strict treatment regulations such as the Medical Service Act and environmental laws. For example, because legal sanctions are imposed on disposing infectious medical waste as general waste, hospital employees must appropriately classify medical waste during the sorting stage to curtail waste generation.

Fifth, the AHP results and the opinions of the three experts indicated a slight difference in the priorities of the five key factors. Thus, healthcare organizations should provide support to front-line employees so that they can freely express their opinions and ideas for performing their medical waste management tasks that are most appropriate for each hospital.

Today, eco-friendly resource management has become important for creating a sustainable green enterprise due to increasing air pollution, climate change, and plastic waste that threaten human health. The global medical waste management market is expected to grow from USD 7.2 billion in 2020 to USD 12.8 billion by 2030 [47]. Thus, anticipating problems that may arise from medical waste generation would be important to all healthcare organizations. The results of this study provide new insights to developing strategic plans for treatment processes and activities to reduce waste.

The theoretical and practical contributions of this study can be summarized as follows. First, our study has broadened the topic and scope of medical waste management by analyzing the priority items that can significantly reduce medical waste generation, unlike previous studies which primarily focused on waste treatment methods. Second, our research method can be applied to other industries that are concerned about reducing waste generation or recycling resources. Finally, the evaluation items identified and analyzed in this study can also be applied to related industries that are struggling to manage waste. Medical waste management approaches may differ among healthcare providers due to their specific characteristics. This study identified and evaluated priority items (factors) that generate medical waste; therefore, the presented results can be used as useful data for developing strategies and policies for medical waste management.

### 5.2. Limitations and Future Research Directions

This study has several limitations. First, due to the small sample size (16), statistical verification for the substitutability index could not be performed. Second, although the amount of data required for AHP was appropriate, the fact that we received only 16 valid responses indicates the difficulties involved in the pairwise comparison for medical staff. Therefore, conducting additional surveys, including a pre-survey training session for respondents, would help collect objective and valid data. Furthermore, future studies should consider broadening the population base, as this study focused only on medical staff at the point of contact in generating medical waste. Third, due to a lack of previous studies on management and treatment activities for reducing medical waste produced by healthcare workers, the evaluation items were developed with a focus on items suggested in waste management research in general and the opinions of healthcare workers in handling medical waste. Future studies should consider the more in-depth development of priority items based on a survey of a broader population of medical personnel. Fourth, the causes and solutions of the medical waste problem were examined by comparing the AHP results with the opinions of three experts. However, because this study selected three experts randomly, it may be prudent to select more objective and representative experts in future studies. Fifth, this study focused on the strategies and activities to minimize medical waste; however, it did not explore other important issues related to medical waste management. For example, optimal economic efficiency and management of medical waste activities are critical topics that need to be researched to secure a sustainable healthcare environment. These are key future research areas of medical waste management. Lastly, because this study was conducted in South Korea, its global generalizability is limited. Therefore, future studies should perform comparisons by analyzing cases from more countries in varying degrees of healthcare services.

## Figures and Tables

**Table 2:** Synopsis of the medical waste stream in Korean hospitals.

Step	Location in the Hospital	Medical Waste Stream	Key Points	Other
0	Storage warehouse of the purchase department		· Purchasing policy · Stock management · National insurance policy	
1	Discharge in care units	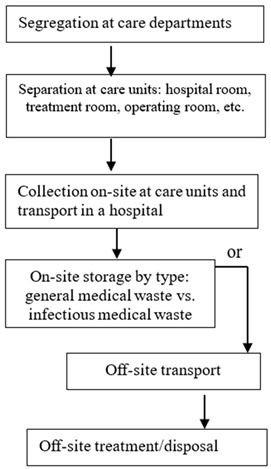	· Appropriate containers for each type of medical waste	
2			· Management to prevent waste from leaking	
3	Storage facility		· Confined bags or containers	· Sanitation worker
4			· Prohibition of reuse of dedicated containers · Adequate storage room · Limited time of keeping	· Environmental technician/ · Infection control department
5	Collection, transportation, and treatment: outside of the treatment facility		· Appropriate vehicles	· Medical waste management by radio-frequency identification
6			· Recycle medical waste · Disposal in sterilization and crushing facilities · Incineration or landfill	

Source: ICRC [18].

**Table 3:** Measurement items for this study.

Factors	Measurement Items
Raising awareness	Recognize the importance of good healthcare waste management
	Setting up a waste management team with responsibility
	Committee to develop waste management plans
	Integration into daily operations
Operational management issues	Operational standards for medical waste
	Prevention plans for potential medical waste treatment problems
	Estimate medical waste management costs
	Develop and implement medical waste management plans
Medical waste management	Safe storage of leak-proof infectious medical waste
	Policies or manuals on separation of medical waste by type
	Regular monitoring to ensure compliance with procedures
	Simple plans to implement medical waste management for staff, including ancillary staff
Environmental assessment	Policy, legal, and administrative framework
	Environmental and health impact monitoring
	Environmental management and training
	Safe medical environment from medical waste
Training for medical waste management procedures	Education on the risks of incorrect medical waste management
	Training on waste separation operations
	Training on staff responsibilities and roles in managing medical waste
	Technical training on the application of waste management practices

**Table 4:** Respondents’ demographic characteristics.

Variables	Categories	N	%	Characteristics	Categories	N	%
Gender	Male	6	37.50	Work experience	Less than 10 years	6	37.50
	Female	10	62.50		More than 10 years	10	62.50
Age	21–30	2	12.50	Knowledge related to medical waste	High	8	50.00
	31–40	8	15.00		Medium	6	37.50
	More than 41	6	37.50		Low	2	12.50
Care units	General ward	4	25.00	Waste management training experience	Yes	14	87.50
	Isolation ward	3	18.75		No	2	12.50
	Emergency room	3	18.75	Importance of medical waste management (multiple responses)	Practice	16	100.00
	Intensive care unit	3	18.75		Attitude	12	75.00
	Operating room	3	18.75		Education training	4	25.00

Total respondents: 16 (100.00%).

**Table 5:** Results of the pairwise comparison matrix.

Level 1			Level 2				
Factors: CR	Importance	Ranking	Measurement Items	Local		Global	
				Importance	Ranking	Importance	Ranking
Raising awareness: 0.116	0.175	4	Recognizing the importance of good healthcare waste management	0.318	1	0.057	7
			Setting up a waste management team with responsibility	0.257	2	0.055	8
			Committee to develop a waste management plan	0.206	4	0.024	19
			Integration into daily operations	0.219	3	0.032	15
Operations management issues: 0.124	0.217	2	Operational standards for medical waste	0.352	1	0.084	3
			Prevention plan for potential medical waste treatment problems	0.124	4	0.026	18
			Estimate medical waste management cost	0.231	3	0.036	14
			Develop and implement a medical waste management plan	0.293	2	0.047	11
Medical waste management: 0.147	0.266	1	Safe storage of secure leak-proof and infectious medical waste	0.334	1	0.091	1
			Policies or manuals on separation of medical waste by type	0.283	2	0.065	5
			Regular monitoring to ensure compliance with procedures	0.179	4	0.048	10
			Simple-to-implement medical waste management for staff	0.204	3	0.087	2
Environmental assessment: 0.094	0.164	5	Policy, legal, and administrative framework	0.175	4	0.021	20
			Environmental and health impact monitoring	0.293	2	0.043	12
			Environmental management and training	0.227	3	0.031	16
			Safe medical environment from medical waste	0.305	1	0.051	9
Training for medical waste management procedures: 0.127	0.178	3	Education on the risks of incorrect medical waste management	0.235	3	0.039	13
			Training on waste separation operations	0.278	2	0.063	6
			Training on staff responsibilities and roles in managing medical waste	0.298	1	0.071	4
			Technical training on the application of waste management practices	0.189	4	0.029	17

**Table 6:** Expert opinions on the analytic hierarchy process (AHP) results.

	Common Perspective	Reason or Cause	Solutions
AHP results	① Of the five items, medical waste management is the first priority item ② Second, training for medical waste management procedures ③ Third, raising awareness ④ Fourth, operational management issues ⑤ Fifth, environmental assessment	〮 Waste management is the most important as waste cannot be re-sorted after placing it in a waste bin. It must be properly sorted at the first time of sorting 〮 Education and training are necessary to minimize the occurrence of legal problems	〮 The importance of medical waste management should be clarified through employee education and training, as well as increased awareness 〮 Laws on medical waste management should be improved
Expert opinion	〮 The problem of waste mixing during waste sorting in separate boxes/bins (infectious and general medical waste) in each treatment unit 〮 The problem caused by an inability to classify waste even after training and education on waste sorting and disposal	〮 The problem of neglecting thorough waste sorting from the initial stage 〮 The problem caused by focusing more on avoiding legal sanctions from infectious medical waste leaks, such as alcohol swabs 〮 Absence of a dedicated organization within the hospital	〮 Continuous and regular education and training on waste management 〮 Subdivided arrangement of waste bins for each treatment room 〮 Active public relations efforts by the relevant department

## Data Availability

The data presented in this study are available on request from the corresponding author.
